# Successful Ligation of the Innominata

**Published:** 1864-11

**Authors:** 


SUCCESSFUL LIGATION OF THE INNOMINATA.—Dr. D. L. Rogers, in a letter to Prof. V. Mott (*Med. Times and Gazette*, Aug. 20, 1864), communicates a brief notice of the successful ligation of the innominata by Dr. A. W. Smith, one of the surgeons of the Charity Hospital, New Orleans.

The subject was a mulatto man, aged 33 years, with a large aneurism. On the 15th of last May, Dr. S. applied a ligature to the arteria innominata and to the right carotid about one inch above the origin. Hemorrhage from the wound recurring, Dr. S., on the 19th of July, ligated the vertebral artery. The patient, it is stated, recovered.—*Am. Jour. of the Med. Sciences.*

